# Successful Ablation of a Left-Sided Accessory Pathway in a Patient with Coronary Sinus Atresia and Arteriovenous Fistula: Clinical and Developmental Insights

**Published:** 2011-03-25

**Authors:** Sandeep M Patel, Christopher J McLeod, Paul A Friedman, XK Liu, Samuel J Asirvatham

**Affiliations:** 1 Department of Internal Medicine; Mayo Clinic, Rochester, Minnesota; 2 Division of Cardiovascular Diseases; Mayo Clinic, Rochester, Minnesota; 3 Department of Pediatrics and Adolescent Medicine - Mayo Clinic, Rochester, Minnesota; 4 Heart Center for Excellence, Kalamazoo, Michigan

**Keywords:** Coronary sinus, coronary AV fistula, accessory pathway, epicardially-derived cells, coronary atresia

## Abstract

**Background and objectives:**

While radiofrequency ablation catheter ablation of accessory pathways is generally safe and effective, anatomic variants can cause considerable challenges in effecting cure.  Our objective was to use an unusual case where coronary sinus was absent and arterial venous fistula was present and a left-sided pathway required mapping and ablation to develop a framework to approach difficult cases.

**Method:**

A detailed literature search and review of contemporary cardiac embryology was undertaken to attempt and to explain a common developmental anomaly.  Adjunctive approaches during the ablation procedure, including intracardiac ultrasound, were used to guide mapping and ablation despite the lack of coronary sinus access.

**Results:**

The accessory pathway was successfully ablated using a transseptal approach and intracardiac ultrasound guided mapping of the mitral annulus.  A potential common mechanism to explain the apparently disparate anatomic variants in this patient was formulated.

**Conclusions:**

Cardiac conduction development is complex and accessory pathway conduction may occur in the setting of arteriovenous anomalies thus providing insights as to the cause of WPW syndrome. Successful mapping and targeted ablation of left-sided pathways may be accomplished even when coronary sinus access is not possible.

## Case

The patient was a 19-year-old pregnant woman who had undergone two prior accessory pathway (AP) ablation attempts at another institution for a symptomatic supraventricular tachycardia. During the initial studies, a left-sided AP was identified, yet cannulation of the coronary sinus (CS) could not be achieved. No ablation was performed, and it was suggested that perhaps a prominent Thebesian valve or maldevelopment of the CS ostium had prevented access.

**Figure 1:**
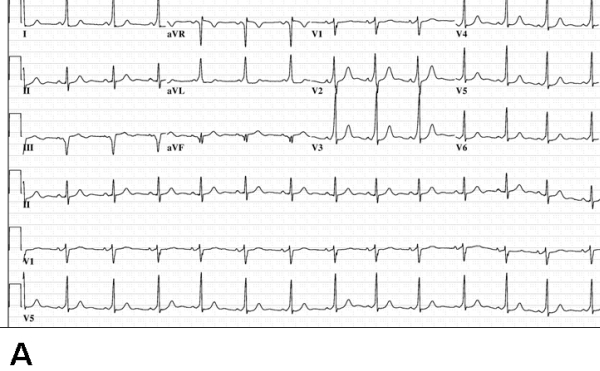

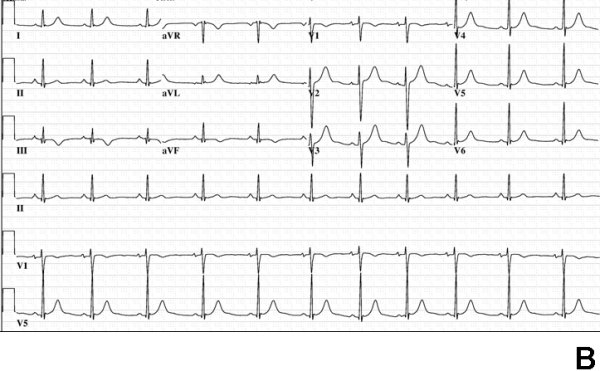
Electrocardiograms at baseline (panel A) and postablation. Baseline ECG shows manifest preexcitation with suggestion of a right-sided pathway (positive delta wave in lead I, S wave larger than R wave in lead V1).  Panel B shows absence of preexcitation following ablation in the left posterior region (see text for details).

**Figure 2:**
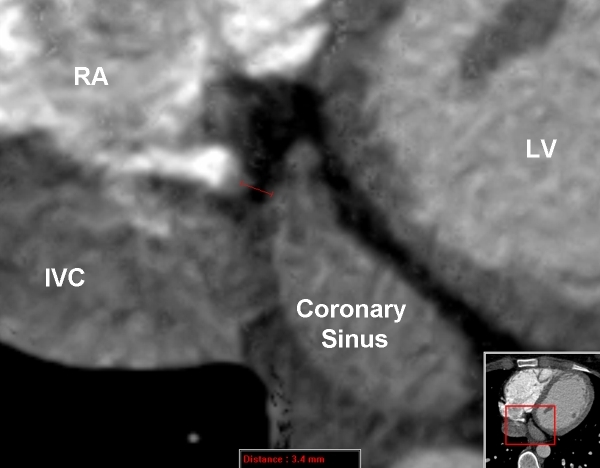
Magnified CT scan image showing coronary sinus ostial atresia. The CS is seen to taper abruptly and end approximate 3.4 mm before reaching the right atrium (see figure 3 for comparison). Abbreviations: RA = right atrium, LV = left ventricle, IVC = inferior vena cava

**Figure 3:**
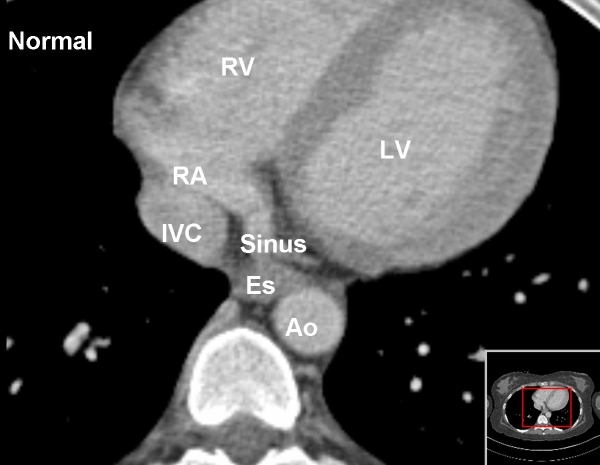
The normal emptying of the coronary sinus into the right atrium is shown for comparison with the abnormality found in this patient (figure 2). RV = right ventricle; AO = aorta, ES = Eustachian Ridge. RA = right atrium, LV = left ventricle, IVC = inferior vena cava

**Figure 4:**
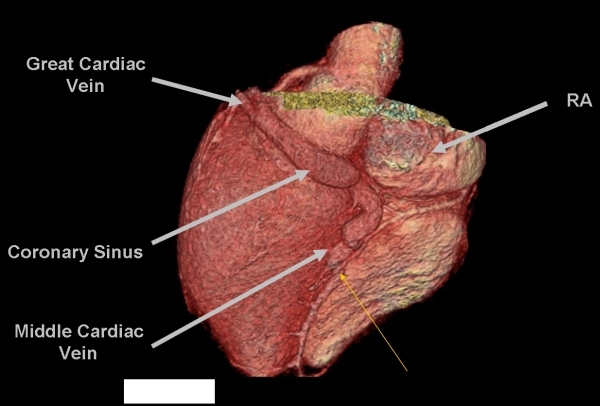
Three-dimensional reconstruction of the posterior and left posterolateral surface of the heart showing the markedly enlarged great cardiac vein and middle cardiac vein in tehis patient with coronary atresia. The thin yellow arrow points to the site of coronary arteriovenous connection between the posterior descending artery and the mid portion of the middle cardiac vein.  Note the abrupt enlargement of the middle cardiac vein proximal to this malformation.

The patient's physical examination was unremarkable, and her electrocardiogram revealed sinus rhythm with a PR interval of 108 ms, a QRS axis of -10 degrees and a QRS duration of 112 ms. The electrocardiogram revealed sinus rhythm with evidence of preexcitation. The delta wave was positive in lead I, isoelectric in leads II and V1 with transition in V2, and negative in lead AVF. The S wave was dominant in lead V1 (Figure 1). Transthoracic echocardiography revealed normal left and right ventricular size and function. To clarify the CS venous anatomy, levo-phase coronary angiography and cardiac CT imaging with 3-D reconstruction was obtained - confirming no direct connection between the CS and the right atrium (Figure 2,3,4). In addition, the middle cardiac and great cardiac veins were dilated and tortuous; venous drainage occurred through small tributaries draining into the right ventricle and right atrium. An arteriovenous communication was also noted between the middle cardiac vein and the posterior descending artery.

Repeat electrophysiological study at our institution confirmed bidirectional left-sided AP conduction. Intracardiac echocardiography was used to interrogate the septum, confirming no clear connection from the CS to the right atrium. After transseptal puncture, further mapping demonstrated earliest conduction on the mitral valve annulus about 0.5cm from the atrial septum and posterior to the plane of the middle cardiac vein. Using an SL4 sheath (Daig Corp., Minnetonka, MN) for better stability and contact, ablation in an annular position was successful. Repeat ECG after the procedure and the following day revealed no preexcitation.  Followup at two years has revealed no recurrent arrhythmias.

## Discussion

We report successful ablation of a left-sided AP in a patient with CS atresia and an arteriovenous malformation. This unique case highlights both the technical difficulties encountered when the CS cannot be cannulated, as well as the aspects common to the development of coronary vessel anomalies and APs [1].

### Epicardially-derived cells (EDCs) and Coronary Venous/Arteriovenous Anomalous Development

Although the precise pathogenesis of coronary arteriovenous fistulae is unknown, Epicardially-derived cell (EDC) and angioblast differentiation are likely involved. Through a vascular endothelial growth factor mediated pathway, EDCs differentiate into angioblasts and subsequently into endothelial precursors. The further development of an endothelial precursor into an artery or vein involves coordinated cellular signaling known as the Notch and Ephrin pathways [2].  Activation of the Notch pathway in an endothelial precursor produces suppression of the venous fate, leading to the formation of an artery. The Ephrin pathway has also been implicated in the development of receptors for arteries and veins: Ephrin-B2 for arteries and Ephrin-B4 for veins. Knockout studies have identified a critical interaction between Ephrin-B2 and Ephrin-B4 [2]. Without this interaction, a proper arteriovenous boundary would not be established, and instead various arteriovenous malformations would occur [2,3]. Thus, arteriovenous anomalous development may coexist with compression or developmental failure of a venous component: in this patient the CS ostium.

### EDCs and AP Development

AP development is such that most are found within the paraseptal regions and consist of strips of myocardium and rarely specialized cells [2,3]. APs are thought to develop secondary to disruption of the discontinuity between the atria and the ventricles [4-7].  The electrical insulation is provided by the fibrous tissues of the AV groove and the hinge lines of the valves. This separation between atrium and ventricle forms as the endocardial cushion tissue forms the scaffolding for the valve leaflets and the annulus fibrosis [2,5].  Once this scaffolding is complete, EDCs migrate through this fibrous network ultimately ensuring the electrical isolation between atria and ventricles. The importance of this critical migration has been well-demonstrated in animal models, and inhibition of EDC migration allows the persistence of broad bundles of accessory atrioventricular canal myocardial connections with concomitant ventricular preexcitation [5]. Thus AP formation and coronary arteriovenous fistulae both rely on intact targeting of the EDC.

### Clinical/Electrophysiological Implications

Ablationists routinely cannulate the CS to map the left atrium and left ventricular annulus. In this patient, the inability to cannulate the CS created difficulty with multiple attempts required to successfully ablate the left-sided pathway. Intracardiac ultrasound guidance for transseptal puncture and for visualizing the annulus (usually defined by the CS catheter) enabled successful ablation [3,8].

In addition to CS atresia, prominent Thebesian valves, anomalous origin for the CS [9], unroofing of the CS, and transposition of the CS ventricle to the tricuspid valve following the repair of Ebstein's anomaly [10,11] should also be considered and similar solutions applied when the CS cannot be cannulated. In addition to intracardiac ultrasound, levo-phase coronary angiography and multi-site CT with 3-D reconstruction were very valuable in this patient to define the underlying anomaly and to guide ablation.

CS ostial atresia associated with arrhythmia [12-14], atrial flutter, AV nodal reentry [15], as well as AP-mediated tachycardia has been described. Thus the knowledge of this potential association when confronted with cannulating the CS should specifically be considered.

### Developmental Implications

The patient in this vignette had three apparently separate cardiac anomalies, namely an AP, CS ostial atresia, and coronary arteriovenous fistula. Can these apparently disparate anomalies be traced to a common embryological fault?

**Figure 5:**
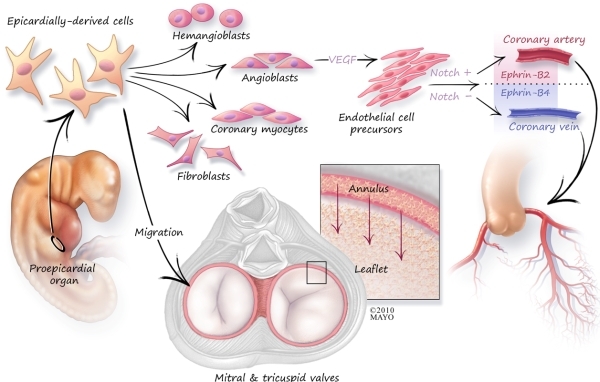
Schematic outline of cardiac embryogenesis. The importance of the epicardially derived cells in conduction system, septation, annulus development, as well as vascular development is described in the text.

During development, specialized mesenchymal cells, known as the proepicardium, transform into clusters of formations, known as the proepicardial organ, which ultimately form cellular bridges [1,7,16]. In turn, these migrate and stratify to give rise to the pericardium and epicardium (Figure 5).  The EDCs are multi-potent, potentially developing into angioblasts, hemangioblasts, myocytes, and fibroblasts - forming the basic building blocks of the coronary vasculature and the conduction pathways.

## Conclusion

This case not only highlights the complexities of ablation in congenital heart disease but also serves as a vehicle for understanding common embryological origins. Although the mechanistic association is surmised - the fundamental role of the EDC in the formation of the coronary vasculature and the conduction system is highlighted. Subtle alterations in EDC signaling not only portend cardinal cardiac structural abnormality, they also provide insight into normal developmental pathways. Epicardial biology is a relatively new field of cardiac developmental biology and further research into the multi-potent EDCs will likely provide us with further insight into the links between conduction abnormalities and congenital heart disease.
